# The Present Issue

**Published:** 1860

**Authors:** 


### The Present Issue.

As will be perceived, we issue under one cover the January and February numbers of the Journal. The explanation of this has been given heretofore. We hope to be able to send out the Journal, hereafter, during the month for which it is designed.

